# Nurses and health care for gay adolescents*

**DOI:** 10.1590/1518-8345.6293.3792

**Published:** 2022-11-07

**Authors:** Luan Sudário Melo, Maria Aparecida Bonelli, José Ricardo de Carvalho Mesquita Ayres, Glauber Weder dos Santos Silva, Flávio Adriano Borges, Monika Wernet

**Affiliations:** 1 Universidade Federal de São Carlos, São Carlos, SP, Brazil.; 2 Universidade de São Paulo, Faculdade de Medicina, São Paulo, SP, Brazil.; 3 Secretaria de Estado da Saúde Pública do Rio Grande do Norte, Natal, RN, Brazil.; 4 Universidade Federal de São Carlos, Departamento de Enfermagem, São Carlos, SP, Brazil.

**Keywords:** Delivery of Health Care, Adolescent, Sexual and Gender Minorities, Nurses, Symbolic Interactionism, Nursing Care

## Abstract

**Objective:**

to analyze nurses’ statements about health care for gay adolescents.

**Method:**

qualitative study, anchored on the Thematic Analysis of Clarke and Braun, with adoption of Symbolic Interactionism as a theoretical framework, since it favors the understanding of the relationship between behaviors, interactions, and social meanings. Twelve nurses recruited using the snowball sampling technique were remotely interviewed via the Google Meet^®^ video-conferencing app.

**Results:**

four themes were elaborated throughout the comprehensive-interpretative process: “Gay adolescents, agendas, and relation with health;” “The gay adolescent’s family and care;” “Relationship with gay adolescents in care,” and “Limits to nursing care for gay adolescents.”

**Conclusion:**

the statements denounce stigmas and symbols derived from cisheteronormativity as intervening in the relationship and indicate the urgency of investing in the intersubjective encounter with gay adolescents and their families in a horizontal, affective, and empathic relationship, with chances of favoring public defense of the right to health. There are comments on the nurses’ attitude and qualification of care for this population.

## Introduction

Sex and gender dissidences, which include lesbian, gay, bisexual, transvestite, transgender, queer, intersex, asexual and other gender variability (LGBTQIA+) people, circumscribe a social field of struggle and power that is antagonistic to compulsory heterocisnormativity (pre-established patterns of gender and sexuality), and are related to the creation of subjective bodies and counter-hegemonic experiences. In the LGBTQIA+ community, gays are male homosexuals, men who challenge machismo and patriarchy, and whose bodies, pleasures, desires, affections, and sociability are experienced with each other. In Brazil, gays represented 1.4% of men over 18 years of age who responded to the National Health Survey (PNS) in 2019^1^.

In the time frame of the second decade of life (10 to 19 years of age), as defined by the World Health Organization (WHO)^2^, a concept also adopted by the Brazilian Ministry of Health, adolescents experience identity processes, changes and transitions in biopsychic issues and social relationships that mobilize understanding, feelings, and emotions^3^ ^-^ ^4^. The condition of being an adolescent and a gay may mean double vulnerability and has repercussions on increased chances of not accessing or not being accepted in the health system due to stigmas.

Primary Health Care (PHC) is described as obstructing the right to health, promoting discrimination, and producing embarrassment for gay adolescents^3^ ^-^ ^4^. Access to the health care system, the identification of needs, and care negligence are pointed out^5^ as barriers to openness and listening^5^ ^)^ and cause adolescents and young people not to seek health services^6^. In this context, the fragility of the bond with health professionals/services is highlighted^6^.

In this context, interpersonal nursing care, guided by the values of human dignity and social justice, appears as having great potential to welcome and provide care for gay adolescents within the PHC^7^. On the other hand, nursing, despite having care in its genesis, may provide a reduced and technical “care,” devoid of relational support^8^ ^-^ ^10^.

Recent literature is scarce and lacking nursing research on gay adolescents. By surveying the main national and international databases, it is possible to observe research on this topic carried out in the United States in the last three years^11^ ^-^ ^26^, with the focus on the nursing care area^12^ ^-^ ^31^. Such pieces of research seek to understand the level of knowledge that nurses/health professionals have of LGBTQIA+ issues^11^ ^-^ ^12^ ^,^ ^14^ ^,^ ^22^ ^-^ ^23^, and adolescents in this group are their main target population^11^ ^,^ ^13^ ^,^ ^15^ ^,^ ^17^ ^,^ ^20^ ^-^ ^26^ ^,^ ^28^ ^,^ ^30^ ^-^ ^31^. These studies are conducted mainly in the school context^16^ ^,^ ^20^ ^-^ ^21^ ^,^ ^25^ ^-^ ^26^ ^,^ ^29^ ^-^ ^31^ and PHC^17^ ^-^ ^19^ ^,^ ^28^, and most of them have been quantitative^11^ ^-^ ^12^ ^,^ ^15^ ^-^ ^16^ ^,^ ^19^ ^,^ ^24^ ^-^ ^25^ ^,^ ^27^ ^,^ ^29^ ^-^ ^30^, when compared to qualitative ones^13^ ^-^ ^14^ ^,^ ^22^ ^-^ ^23^ ^,^ ^31^.

In line with the above and the guidelines of the National Policy for Comprehensive Health of Lesbians, Gays, Bisexuals and Transgender People (PNSI-LGBT) for care and research qualification within PHC or Specialized Care (SC) for the LGBTQIA+ population, one sought to fill, to some extent, the epistemological and methodological gap of the national production in nursing regarding care for gay adolescents. It is assumed that nurses take care of gay adolescents and that they can contribute to the composition of human and social rights related to care for this population and, therefore, it is interesting to analyze these care relationships. Thus, this study aims to analyze nurses’ statements about health care for gay adolescents.

## Method

### Study type

This is a qualitative study, guided by the Consolidated Criteria for Reporting Qualitative Research (COREQ) and anchored on the Thematic Analysis of Clarke and Braun^32^ ^-^ ^33^, taking Symbolic Interactionism (SI) as a theoretical framework, since it favors the understanding of the relationship between behaviors, interactions, and social meanings^32^.

### Setting

The study was developed in the context of PHC (Family Health Strategy) and SC/LGBTQIA+ (Specialized Health Care for LGBTQIA+/LGBTQIA+ Health Outpatient Clinics) in the five regions of Brazil, covering eight cities (São Carlos, Ibaté, Paulínia and São Paulo, state of São Paulo; Florianópolis, state of Santa Catarina; Rondonópolis, state of Mato Grosso; Aracaju, state of Sergipe; and Belém, state of Pará). All the services were provided in urban areas and offered care for the gay population.

### Participants

Twelve nurses participated in the study, eight working in PHC and four in LGBTQIA+ SC. The snowball sampling technique was used to recruit the nurses, recommended for situations of restrictions on identifying and inviting participants^34^. The technique favors the exponential chain of indications from participants^34^. In the zero wave of recruitment, two participants (seeds) were included to start the referral chain, randomly selected after dissemination of the research on social networks (WhatsApp^®^, Facebook^®^, Instagram^®^, Twitter^®^), via cards - informative texts and invitations to participate -, where the candidate was asked to contact one of the researchers. The seeds were encouraged to refer or invite new candidates in other cities and states of the Federation, which resulted in the enrollment of 12 nurses. The final number of participants was defined according to the sufficiency criterion assigned by the authors, based on the understanding reached about the object of interest^35^.

None of the interviewees had a relationship with the authors. All nurses have been previously presented with the study by the first author, and no one refused to participate.

### Selection criteria

For inclusion in the study, participants should: be a nurse and working in the Health Care Network (RAS) of the Unified Health System (SUS), within PHC or in SC/LGBTQIA+; have professional experience of more than 1 year, considered to be enough time to create bonds with the community, and declare to have experience as a health professional in care for gay adolescents. Candidates who were on maternity leave, vacation, and any other type of absence from work were excluded.

### Data collection

The interviews were scheduled after previous contact with the candidates and eligibility criteria evaluation by the researchers. The empirical elements were collected by the main researcher based on a sociodemographic questionnaire with closed questions and an online interview, with open questions and an average duration of 35 minutes, from February to June 2021. Conducting online interviews favored geographic coverage of different Brazilian regions, secure data archiving, low cost, minimization of group influence effect, and possible anonymity. Nevertheless, impossibility of collecting non-verbal data and risks of not getting in-depth answers^35^ are considered challenges to the study.

The interviews, recorded on the Google Meet^®^ video-conferencing app and stored on a hard drive reserved for this purpose, began with questions of sociodemographic characterization, to then explore the study focus from the request: “Tell me how you care for the gay adolescent in your daily work”. Questions articulated with what was being exposed were presented throughout the interview to broaden understanding and more details.

### Data analysis and treatment

As data were collected, a Microsoft Office Word^®^ spreadsheet was filled out with sociodemographic data, and integrity, duplicity, and completeness were verified. Data were analyzed using simple descriptive statistics.

The data extracted from the interviews were transcribed and organized using Microsoft Office Word^®^ from detection and correction of linguistic errors, when vocabulary, grammar, and language vices were revised. Statement content analysis started during transcription, by writing descriptive memos that supported coding and establishment of the themes. Then, the interviews were analyzed systematically from the following steps: reiterative readings of the interview transcriptions for familiarization, highlighting excerpts that were later taken for coding; grouping of codes in order to generate initial themes from the central construct, and articulation of the elements that composed it^32^ ^-^ ^33^.

With the data analysis completed, one sought to validate the themes and subthemes of the results with two of the interviewees, one from PHC and the other from SC, invited by email. They received the analytical themes description with their respective statement fragments and answered the following question: “Do the data represent the reality of everyday life in care for gay adolescents in PHC and in SC/LGBT+?” The feedbacks received validated the results.

### Ethical aspects

The study followed the recommendations of the resolutions in force in Brazil for research involving human beings, was appreciated and approved by the Ethics Committee in Research with Human Beings, with favorable opinion No. 4.560.347 of February 25, 2021 and CAAE registration: 40210520.30000.5504. To preserve participants’ anonymity, the fragments of the interviews were identified as “n.PHC” for PHC nurses and “n.SC” for SC/LGBTQIA+ nurses (ordinal number indicates the order of entry in the study). The Informed Consent Form (ICF) was signed via Google Forms^®^ and a copy was made available to the participant via email.

## Results

### Participants’ profile

**Figure 1:**
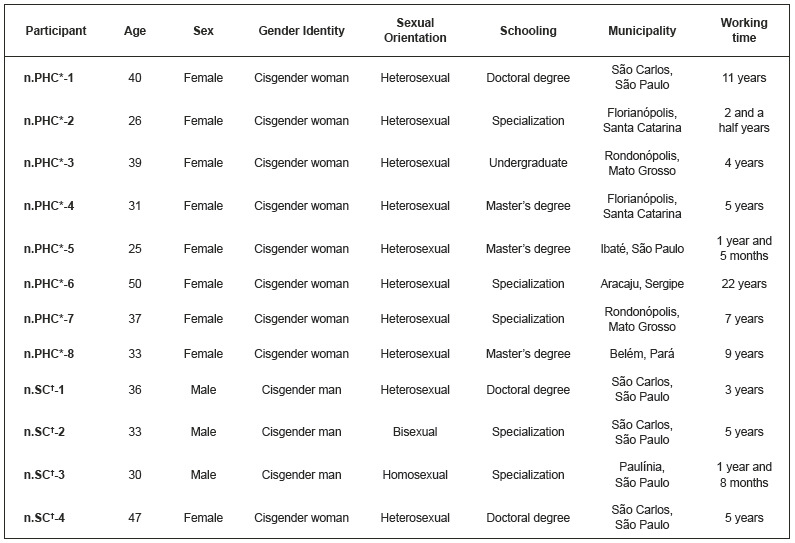
Study participants’ profile *n.PHC = Primary Health Care; ^†^n.SC = Specialized Care

Twelve nurses participated in the study, nine women and three men, all with cisgender identity, 10 identified as heterosexual, and the mean age was of 36 years. They worked mainly in PHC (67%) and their professional experience ranged from 1 year and 5 months to 22 years; most attended postgraduate studies (Figure 1).

Four themes were elaborated throughout the comprehensive-interpretative process: “Gay adolescents, agendas, and relation with health;” “The gay adolescent’s family and care;” “Relationship with gay adolescents in care,” and “Limits to nursing care for gay adolescents.”

#### Gay adolescents, agendas, and relation with health

Adolescents were defined as a population little connected with health services compared to other population groups. The nurses reported that when the adolescents are gay, not seeking the service is even more significant and that what prevails is the situation where they look for the health care service because of issues related to sexuality, sexually transmitted infections (STIs), use of illicit substances, mental/psychic health, and violence. The behavior of not seeking the health service was perceived as a result of fear of prejudice and judgment by health professionals and service users: *I think the main resistance of this population* [gay adolescents] *is* [because of] *the fear of judgment. For thinking they will be there waiting and the users who are also waiting for an appointment will look at them differently. This younger population, they don’t have many other complaints, their complaints are more focused on their sexual life, on emotional issues, because there are very few who have some other comorbidity, some condition* (n.PHC-7).

The association between adolescence, novelties, and discoveries was evidenced. Despite being mentioned as of different types, the nurses exemplified and emphasized the issues of sexuality. According to the interviewees, gay adolescents emphasize risks and highlight the presence of internalized judgments and guilt that remain in self-reflection and result in suffering: *And I think that adolescence is a very troubled moment, you are getting to know yourself, you are in the process of more intense social interaction, hormonal and physical changes, everything is very new, there are uncertainties, I don’t know, it’s a kind of crazy (n.PHC-5).* […] *and who said that he* [gay adolescent] *doesn’t blame himself for having sex without a condom, for having suffered a sexual assault, for having abused a certain substance, for having perceived himself* [as gay]*, in short, placed himself in situations of vulnerability* [?](n.SC-2)*.*

They also associate the adolescent’s mental health with difficulties in dealing with suffering, especially in the face of insufficient social support, including the family: […] *he* [adolescent] *has a lot of doubts about what his sexuality is, what the construction of gender is, and, sometimes, he has very practical difficulties, I mean, material ones, he’s homeless, he’s been kicked out of the house, he has no job. Sometimes he has some associated anxiety, some associated psychiatric disorder, and then the person wants to talk about it* (n.SC-2).

#### The gay adolescent’s family and care

The family was considered a difficult point to be addressed in the context of care for gay adolescents, as it does not accept effectively the sexual orientation assumed by the adolescent, despite being aware of it. They realized that there was concealment and few families spoke openly about the subject: *In my experience, the family is very introspective, it is very difficult to speak to them.* […] *it’s all very delicate, things are not said. The reception is very reluctant, they know* [when the family knows about the child’s sexual orientation]*, they realize everything, they coexistence with their child, with their niece, with their grandson, but everything is still very veiled* (n.PHC-6)*. I think working with gay adolescents is very difficult, mainly because of family interference.* […] *because the father doesn’t want to talk about it, and it also causes discomfort for the boy who doesn’t want us talking with his father about it because, sometimes, the father doesn’t even know it, you see?* (n.PHC-5).

Regarding the family’s participation in care, the difficulty of open and clear dialogue about sexual and sexuality issues was identified as restricting the nurse-adolescent interaction. The professional’s understanding is that care for this adolescent is enhanced in the absence of parents/family. Despite this, when they are present, they find it difficult to ask to be alone with the adolescent: *Sometimes I say to the family: “I’d like to talk to him alone.” And the family asks me: “but why do you want to talk to him alone?” You see? I think it’s complicated, it’s a situation that I still have difficulty dealing with, because when the teenager is there with me and we talk, we exchange some good ideas, but how can we do it? The family restricts this relationship* (n.PHC-5).

For the nurses in this study, the impression that prevails is that, most of the time, the adolescent looks for the service without the family. On the other hand, care experiences were perceived as easier when the family presence means understanding and backup, representing a potential support resource. Even so, when family members are present, nurses adopt a zealous attitude to understand how the family treats the patient, in order for the nursing team to maintain the relation with the adolescent and the family: […] *most of the time, the connection with the family has to be made very, very carefully, very carefully, slowly and calmly, because otherwise we lose the teenager, and the family is lost,* […] *so, many times, we can even lose the family. So, this work has to be very careful* (n.PHC-3).

#### Relationship with gay adolescents in care

Investing in the relationship with the adolescent was presented as structuring for care, and trust and openness to sharing experiences were actively established in it. The nurses realized that prejudice and judgments experienced by gay adolescents in society also crossed the interactions with them in the health services.

The professionals evidenced the reach of a relationship conducive to care when the adolescent started to talk openly and richly about himself: […] *I realize that I’m managing to construct* [care] *or at least I have a clue when the teenager can talk freely about himself, he can talk to me about himself, which is good for him* (n.PHC-2).

The subthemes “Prejudice and establishment of relationships” and “Visibility for care intention” translate understandings and behaviors to develop relationships conducive to care.

#### Prejudice and establishment of relationships

The way of being with the adolescents determined the creation of a bond and context for care. To this end, the nurses bet on respectful and non-invasive interactions, taking care not to generate discomfort, not to reiterate prejudice and judgments, and for the adolescents not to be taken as “different.” These relational beacons contributed to the adolescent revealing himself and the development of a reciprocal relationship: *I try to treat it in the most natural way it is* […]*. And it is a two-way bond, because it’s no use wanting to have this bond with the professional and the professional not allowing it* […] (n.PHC-7)*. I think those people may suffer strong prejudice. I say “may suffer” because I am not part of that group, so, I imagine, I imagine what the other suffers. I try to bring relief, show in the relationship with him that I’m not like that* […] (n.PHC-1).

Interviewees stressed that focusing on the gay adolescent label for the construction of care is not productive, but the attention has to be directed at the needs presented by them. They also mentioned that looking from differences, typifications and judgments is unfavorable to develop intersubjective relationships: *We seek to identify needs through qualified listening, the needs of this individual, what motivated him to look for the service. You need to find the teenager, you know? And you don’t have to think that he is gay, that he can do this or that because he is gay. Create that bond and really care* [for him] (n.SC-1). 

Care presented itself as derived from a shared process. The participants undertook efforts to provide the information that the nurse is a professional who respects, does not judge and intends to welcome them, a professional they can count on.

#### Visibility for care intention

The interviewees were concerned about transmitting the message that they were focused on the adolescent’s needs. They sought to be careful in approaching agendas known as nuclei of prejudice, such as sexuality, sexual relations, and gender identity, and mentioned the gay adolescent’s fears of talking about them. Some of the interviewees, when dealing with these nuclei, established parallels between the behavior of gays and straight adolescents: *I feel that there is still a lot of resistance, the fear of judgment, they are afraid of being judged for some things, for attitudes, if* [the adolescent] *were a straight person, he would not have this fear of coming and talking to me, exposing the sexual life* (n.PHC-7). […] *it’s important to be based on what that person tells me, but, yes, there are some particularities, we have to think about this population, because they do experience other processes in society, which have to be considered* (n.PHC-5).

Interviewees mentioned a unique care, with openness to listening to the adolescent and his needs. To this end, they sought to identify nuclei of suffering, which they believed to lie in issues related to sexual orientation and practices: […] *we are doing this work, which is very unique, for each teenager, for each situation, but in general the reasons end up being very similar, it’s not difficult realize, well, a very, very different issue where that suffering come from, how that is being built. There are a lot of sexual orientation issues* (n.SC-3).

Two gay interviewees conceived the identity proximity and the lived experiences as favorable to the relationship with these adolescents. They perceived in themselves a sensitivity that enabled the establishment of empathy*: I have issues that are LGBT issues, we kind of see in that teenager the teenagers we were and we try to search for alternatives so that these teenagers don’t experience the bad things we’ve experienced, so I think it’s super cool* (n.SC-3).

#### Limits to nursing care

The interviewees’ selves were mobilized in the reflection on their professional behavior in health practices with gay adolescents. They recognized gaps in care, especially in terms of listening and reception, and criticized the work process and professional training: *Many times an 18-year-old person, a 19-year-old person is diagnosed with HIV and then we look at the medical record and observe that this person has already been there two or three times, he has been there about three times and we were unable to have any strategy or any, any kind of service, anything that would prevent the person from becoming infected, for example, with HIV.* […] *Our focus is on the treat and street philosophy, it is still biological* [silence]*. Does any training lead you to think differently? No? Everything that comes from guidance refers to the perspective of illness, of deviation* (n.SC-1).

They also commented on the School Health Program (PSE), recognizing the tendency of ready and prescriptive guidelines, especially in relation to behaviors seen by society as risky to adolescents, such as unprotected sex, pregnancy, violence, and drugs: *And via PSE* [Programa Saúde na Escola] *we talk about sex, drugs, the ‘don’t do it.’ How is the teenager asked to come to us? He is not! We are the ones who do it, or if they* [the school] *ask us to do it, we go there with this purpose* (n.SC-4).

When reflecting on care practices, the interviewees identified in themselves sensitivity to the care agenda for gay adolescents, but also the lack of knowledge, guidelines, and experiences. They denounced structural and ideological barriers anchored on cisheteronormativity, promoting professional detachment from this population, jeopardizing care and the Care Network (RA): […] *but, strategy for seeking those people, I don’t know, I don’t have it. Yeah, we try to do some things. I think they face big obstacles.* […] *The care is very superficial, general. In the end, they are not cared for or little cared, in a poor manner* (n.PHC-5)*. We identified many issues that are LGBT people’s rights being passed over in favor of cisgender and heterosexual issues* (n.SC-3)*. I think there are several challenges that come before the services themselves, there is a service network that cannot absorb these teenagers’ demands* […]*. It’s quite difficult to build a referral network, it’s quite difficult to make proposals together with professionals from other health services* (n.SC-2)*.*

Nurses also revealed incipient or lack of training in the subject, considering it essential for practical transformations in care for gay adolescents: *So, I also think there’s a lot of difficulty, because, like I said, no, no, I’ve never had contact* [with the topic] *in the undergraduate course, in the graduate program I had a subject dealing with sexuality, but it did so generally, sexuality, gender. All this was covered, but in a very brief manner* (n.PHC-2)*.*

## Discussion

The nurse is the professional usually involved in care for gay adolescents, and the relationships established with him and his family affects how this care will be provided^36^. This study found that nurses want to establish interactions favorable to dialogue, demonstration of needs and that enable the adolescents to talk about themselves. Suffering considered particular to this population was highlighted, attributed to prejudice and social judgments. In view of this, the professionals appeared to demonstrate compassion, an element that can favor the encounter and also be a driver of care, contributing to fluid and appropriate relationships^37^.

The results showed an intention to alleviate suffering, but without a description of a significant and unique reach in care interactions. There were intentions to understand the uniqueness of the adolescents’ situation, as well as strategies to increase the understanding of life and other health issues. They denounced *a priori* representations related to homosexuality that obscured and/or neglected needs. Stigmas acted as relational obstacles to the provision of care and, when perceived by gay adolescents, generated uncertainties and ambivalence^38^. The action of interacting involved assuming the role of the other and triggering processes in the adolescent’s self.

The lack of attention to the gay adolescents’ individuality and the professional’s heteronormative behavior drove the adolescent away from the service and professionals^39^, an aspect highlighted by the interviewees. A Spanish study revealed that LGBTQIA+ people described discriminatory attitudes by health professionals, as well as their distrust and fear in this scenario^28^. The deconstruction of heteronormative symbolism is urgent and necessary for the renewal of relational possibilities, a structural element of care, in addition to challenging nurses and other professionals. The professional’s action demonstrative of empathy, solicitude, and compassion regarding the relationship favored the identification of particular needs for each care situation.

The results revealed as fragility the consideration of gay adolescents’ historicity and life context. The basis of nursing practice is the ability to feel a person’s needs through the “I-You” relationship. In the absence of personal experiences and/or arising from situations shared with gay adolescents, care practices tend to be based on general and generalizable knowledge, a limiting aspect for the fulfillment of unique needs^40^.

Stereotypes and stigmas crossed our results. This is visible when, for example, being gay is almost immediately associated with STIs, psychological distress, and family issues. These stereotypes go in the opposite direction of the openness to the other presupposed in the effectiveness of a care encounter.

STIs are on the agenda claimed for the health care for homosexuals; therefore, there is sense and meaning in considering them. One verified criticism of how this symbol directs the professional in care, with reduced opportunities to reveal needs and relational quality^41^. There is a danger of restricting the care service agenda to STIs, especially due to the tendency of stigma intersectionality when non-normative sexuality is present in the care scene^42^. It is urgent to break with the care protocol tendency and the valuation of social labels in its provision, in order for the particular to emerge in and from the relationship.

Another point highlighted was related to the adolescents seeking privacy in health appointments and confidentiality of the information provided there, elements reiterated in the literature, added to the relevance of listening and establishment of a reference professional^43^. In this context, being accompanied by family members is perceived as an obstacle because it generates discomfort and does not allow the adolescent to reveal himself^44^, perception supported by the participants of this study. Depending on the relationship between these adolescents and their families, this aspect may or may not contribute to the care for gay adolescents^45^, an important focus of attention for nurses.

Discovering and coming out as homosexual to the family can lead to fear, guilt, and repression^46^. In addition, the family context is linked to violence against this population^47^. Thus, considering the family in the establishment of support for gay adolescents is essential, with emphasis on the effects that the cisheteronormative model and homophobia can have on family relationships^36^. Given these considerations, the adolescent’s family is also a health care demander, beyond a companion or a probable support^48^. Knowledge derived from family nursing is useful for nurses to assess and intervene with these families^49^. (Not)including family members can promote interactional discontinuity for care (adolescent, family, and professional), weakening its practical success.

Family resistance to approaching gender and sexual orientation with adolescents can act as a determinant for the refusal of gender diversity, favoring a social context that reinforces, reiterates, and leads to prejudice and violence^47^. In turn, nurses and professionals themselves are social actors who act in the opposition or reinforcement of such symbols from their actions.

The interviewees denoted discomfort in addressing issues related to care involving gender identity and sexuality. This difficulty is linked to the lack of understanding, knowledge, and preparation necessary for the incorporation of the LGBTQIA+ culture in care, which are not present in health training^11^. Continuing education related to care for LGBTQIA+ adolescents expanded knowledge and qualified nurses’ behaviors^12^.

Partnerships between different professionals and sectors are relevant for the integrality of care, and the school emerges as strategic for the adolescent population. The PSE is anchored in Health Promotion, which is aligned with the recognition of the space of education as valuable to promote reflections and new thoughts about life and health issues^50^. Thus, it is up to nurses to seek insertions anchored on the PSE and propose discussions related to gender, gender identity and sexuality in the thematic agenda, enabling dialogues perceptions to be exposed^28^. Health actions at school are identified as vertical, disconnected from the school curriculum, supported by a medicalizing paradigm^51^ and cisheteronormative perspectives of little contribution to behavioral sensitization and welcoming diversities.

In view of the above, the way in which professionals incorporate protocols and guidelines from guiding documents in care for gay adolescents needs to be reviewed, providing the effective reception of adolescents and their families. Comprehensive efforts to understand and support the identity process of gay adolescents in the particularity of their social context should be on the agenda of health services and the centrality of care. The bond is created and strengthened depending on the interaction and is related to the transformation of practices to fulfill the adolescents’ health needs^52^. Comprehensive care requires valuing subjectivities, betting on interdisciplinarity and intersectorality^53^.

The incipience and weaknesses that nurses carry to care for this population, which has double vulnerability, that is, being teenagers and gays, may be more related to *how* one educates than *about what* one educates. Reproduction of protocols in teaching without betting on the encounter and effective exposure to the experience of those who demand care reduce perspectives and sensitivity. It is necessary to investigate the validity of the knowledge that emerges and directs the intersubjective relationships between nurses-care demanders^54^. There is no doubt that nurses need knowledge and skills necessary to act safely and competently, but to gain representation as care, practical success, it is necessary to put oneself in the other’s shoes, and professional training leaves much to be desired in this equation.

Few studies address specifically one of the populations represented by each letter of the initialism LGBTQIA+, with a tendency to be approached in a generalized way, which weakens evidence and discussions about the specifics of each population. This study advanced in this direction and contributed with notes related to determinants of relational insufficiency between nurses and gay adolescents and, consequently, weaknesses in the reception of the latter, subjects of rights, worthy of experiencing equity in health. Signals for practical advances were made, especially regarding the influences of prejudice and previous judgments derived from cisheteronormative socialization.

Although the number of study participants can be considered a limitation, their profiles are diversified, and there was great rigor regarding the indicatives of the references listed in the investigation. Data collected remotely may have reduced the chances of clarifying and expanding the data collection and detailing. The evidence, however, is consistent regarding the objective of the study, and the categories developed encompass elements that can be analyzed and incorporated in nursing care for gay adolescents.

## Conclusion

Nurses revealed a tendency to de-subjectify gay adolescents due to the objectivity with which they conducted care and the cisheteronormative stigmas and attitudes that influenced meanings and behaviors. It is extremely important that nurses enable gay adolescents to express themselves, their life history, and needs. Supporting and welcoming their expression and protagonism depends on the nurse’s attitude towards the adolescent and his family, when insufficiencies in terms of empathy and solicitude were present and weakened the process. The professional’s legitimate interest in the gay adolescent and the qualified and stereotype-free listening seemed to be a necessary way to effectively take care of their health.
